# Hospital admission and mortality rates for ischemic heart disease in Thailand: 2012–2021

**DOI:** 10.1186/s13104-024-06803-x

**Published:** 2024-05-19

**Authors:** Boonsub Sakboonyarat, Ram Rangsin

**Affiliations:** Department of Military and Community Medicine, Phramongkutklao College of Medicine, Bangkok, 10400 Thailand

**Keywords:** Ischemic heart disease, Epidemiology, Hospital admission rate, Morality rate, Thailand

## Abstract

**Objective:**

To provide an update on the epidemiology of ischemic heart disease (IHD), including the age-standardized rates of hospital admission and mortality for IHD in Thailand from 2012 to 2021, using the Ministry of Public Health National database.

**Results:**

The overall age-standardized hospital admission rate for IHD decreased from 427.5 per 100,000 people in 2012 to 390.5 per 100,000 in 2021. In men, the age-standardized hospital admission rate was 462.7 per 100,000 people in 2012, reaching 485.8 per 100,000 in 2021, *p* for trend = 0.141. In women, the age-standardization hospital admission rate for IHD dropped by 24.1% over the decade (*p* for trend = 0.008). The overall age-standardized IHD mortality rate in 2012 was 23.4 per 100,000 people, peaked at 28.6 per 100,000 in 2016, and reached 26.9 per 100,000 in 2021, *p* for trend = 0.181. In men, the age-standardized IHD mortality rate rose by 26.6% over a decade, *p *for trend = 0.004. The age-standardized IHD mortality rate among women was consistent from 2012 to 2021, *p* for trend = 0.130. However, for people aged < 60, rising trends in IHD mortality rates over a decade were observed; it rose by 59.6% in men and 36.1% in women.

**Supplementary Information:**

The online version contains supplementary material available at 10.1186/s13104-024-06803-x.

## Introduction

Cardiovascular diseases (CVDs) represent the majority of noncommunicable diseases, accounting for about one-third [1]. The Global Burden of Disease (GBD) study revealed that ischemic heart disease (IHD) is the leading cause of CVD death worldwide, including in Thailand [2]. Regarding the GBD study, the estimated IHD mortality between 1990 and 2017 indicated a decrease in age-standardized IHD mortality by 30.0%, 18.9%, and 53.2% in global, Southeast Asia, and Thailand, respectively [5, 6].

In the past decade, it has been observed that the risk factors for CVD, such as high blood pressure (BP), blood glucose, and obesity, have been increasing among the Thai population [7–13]. According to the National Health Examination Survey (NHES) in 2019, 25.4% of Thai adults have hypertension (HTN), and 9.5% have diabetes [9]. Moreover, the prevalence of obesity has increased by approximately 10% in men and 6% in women over a decade [7–9]. However, there is no updated study about IHD in Thailand. A better understanding of the IHD situation among the Thai population can help policymakers develop targeted interventions to reduce the risk of CVD in Thailand. Therefore, we aim to provide an update on the epidemiology of IHD, including the age-standardized rates of hospital admission and mortality for IHD in Thailand from 2012 to 2021, using the Ministry of Public Health (MoPH) National database.

## Methods

### Data source

We aimed to establish comprehensive national hospital admission and mortality rates for IHD in Thailand. The study analyzed data from two reports: the annual national reports on illness [14–23] and the annual statistics report on public health [24–33] from 2012 to 2021. Since 2012, the Central Office for Healthcare Information has been collecting information on Thai patients' visits to the hospital, both outpatients and inpatients to both public and private hospitals. The Health Data and Information Unit, Strategy and Planning Division of the MoPH reports information on the illness of Thai patients in the annual national reports of illness [14–23], which consist of the cause of illness reported using the International Statistical Classification of Diseases and Related Health Problems, tent revision (ICD-10) codes [34]. Thailand has maintained a record of its death statistics since 1950. The MoPH obtains mortality data from the database of the death certificates issued by the Ministry of Interior, which report the causes of death using the ICD-10 codes. These data are then compiled and included in Thailand's statistics reports of mortality, which are disseminated to the public through the annual statistics report of public health [24–33]. We analyzed the data obtained from these sources to determine trends in IHD-related hospital admissions and mortality rates in Thailand over the recent decade. The Institutional Review Board, Royal Thai Army Medical Department in Thailand, reviewed and approved the study (S012h/67_exp).

### Definition of ischemic heart disease

In the present study, the identification of IHD based on the ICD-10 codes I20-I25, which includes a range of conditions such as I20 Angina pectoris, I21 Acute myocardial infarction, I22 Subsequent myocardial infarction, I23 Certain current complications following acute myocardial infarction, I24 Other acute IHD, and I25 Chronic IHD [34]. The inpatients who were diagnosed with these ICD-10 codes, whether as the principal diagnosis or as a comorbidity, were considered hospital admissions for IHD. Moreover, the IHD mortality was defined based on the appearance of I20–I25 codes in the death certificates. It is worth noting that global and national reports, such as the GBD Study of 2017 and The Heart Disease and Stroke Statistics: A Report of US and Global Data from the American Heart Association [2, 5, 35], have recognized the ICD-10 codes I20–I25 as a standard for diagnosing for IHD and determining the cause of death on the death certificates.

### Data collection process and analysis

The number of inpatients with IHD and deaths associated with IHD, recorded from 2012 to 2021, were retrieved from the annual national reports on illness [14–23] and the annual statistics report on public health [24–33]. The crude hospital admission rate for IHD was determined by dividing the number of inpatients with IHD identified by the size of the mid-year population for the corresponding year (total Thai population). The crude IHD mortality rate was determined by dividing the number of deaths associated with IHD identified by the size of the mid-year population for the corresponding year. The annual Thai population size used in the analysis was based on the mid-year population from the Population and Housing Census reports, National Statistical Office. The hospital admission and mortality rates were expressed as per 100,000 people. All rates were standardized by age adjustment to the 2012 Thai Census, as the ratio of the aged population was less than 60 years and ≥ 60 years in 2012. Hospital admission and mortality rate trends from 2012 to 2021 were tested using a general linear model. A two-sided *p*-value less than 0.05 was considered statistically significant. All statistical analyses were performed using Stata, Version 17 (StataCorp. 2021, College Station, TX).

## Results

**Fig. 1:**
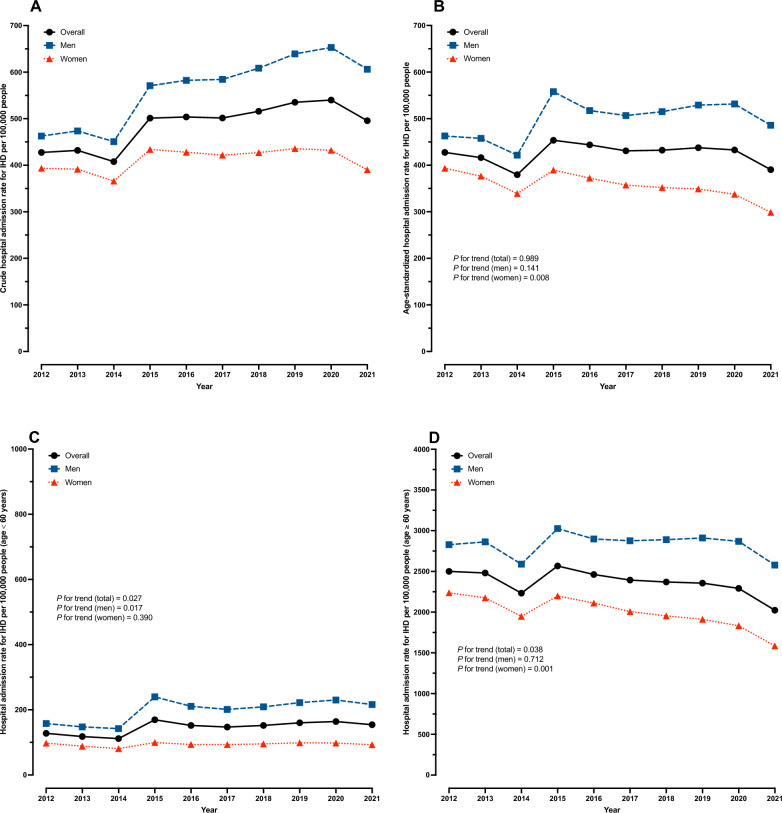
Trends in hospital admission for IHD (per 100,000 people) in Thailand from 2012 to 2021, (**A**) crude hospital admission rate, (**B**) age-standardized hospital admission rate, (**C**) hospital admission rate in people aged < 60 years, (**D**) hospital admission rate in people aged ≥ 60 years

The total population of Thailand has seen a steady increase from 64,266,365 in 2012 to 65,212,951 in 2021. The percentage of people aged 60 and over was 12.6% in 2012 and 18.3% in 2021, as per Table 1. After standardizing for age distribution in 2012, the overall age-standardized hospital admission rate for IHD was consistent at 427.5 per 100,000 people in 2012 and 390.5 per 100,000 people in 2021, *p* for trend = 0.989. In men, the age-standardized hospital admission rate for IHD peaked at 557.8 per 100,000 people in 2015 and reached 485.8 per 100,000 in 2021, *p* for trend = 0.141. On the other hand, a significant decrease in the age-standardization hospital admission rate for IHD in women was observed, dropping by 24.1% over the decade (*p* for trend = 0.008), as shown in Fig. 1. Hospital admission rates for IHD among Thai people below 60 years increased significantly from 2012 to 2021 (*p* for trend = 0.027), with a 36.9% rise observed in men. However, for Thai people aged 60 and above, a decreasing trend was observed during the same period, *p* for trend = 0.038 (Supplementary Table 1).

**Table 1:** Age-standardized hospital admission and mortality rates for ischemic heart disease (IHD) in Thailand from 2012 to 2021

Year	Total population*	Ratio Men: Women	% Aged ≥ 60 years	Age-standardized hospital admission rate for IHD per 100,000 people			Age-standardized IHD mortality rate per 100,000 people		
				Overall	Men	Women	Overall	Men	Women
2012	64,266,365	0.97	12.6	427.5	462.7	393.5	23.4	28.2	18.8
2013	64,621,302	0.97	13.3	416.4	457.6	376.6	26.0	31.4	20.8
2014	64,955,313	0.97	14.0	379.6	421.6	339.0	26.1	31.8	20.5
2015	65,027,401	0.96	14.6	453.5	557.8	389.5	27.2	33.3	21.2
2016	65,013,495	0.96	15.2	443.8	517.5	372.3	28.6	35.6	21.8
2017	65,204,797	0.96	15.8	430.9	506.6	357.4	27.6	35.0	20.5
2018	65,406,320	0.96	16.4	432.2	515.1	352.0	27.0	34.4	19.8
2019	65,557,054	0.96	17.1	437.5	529.0	349.0	26.1	34.0	18.4
2020	65,421,139	0.96	17.7	432.8	531.4	337.6	26.6	34.8	18.7
2021	65,212,951	0.95	18.3	390.5	485.8	298.8	26.9	35.7	18.3
Percentage change, 2012–21				− 8.7%	5.0%	− 24.1%	15.0%	26.6%	− 2.7%
*P* for trend				0.989	0.141	0.008	0.181	0.004	0.130

*Number of mid-year population

**Fig. 2:**
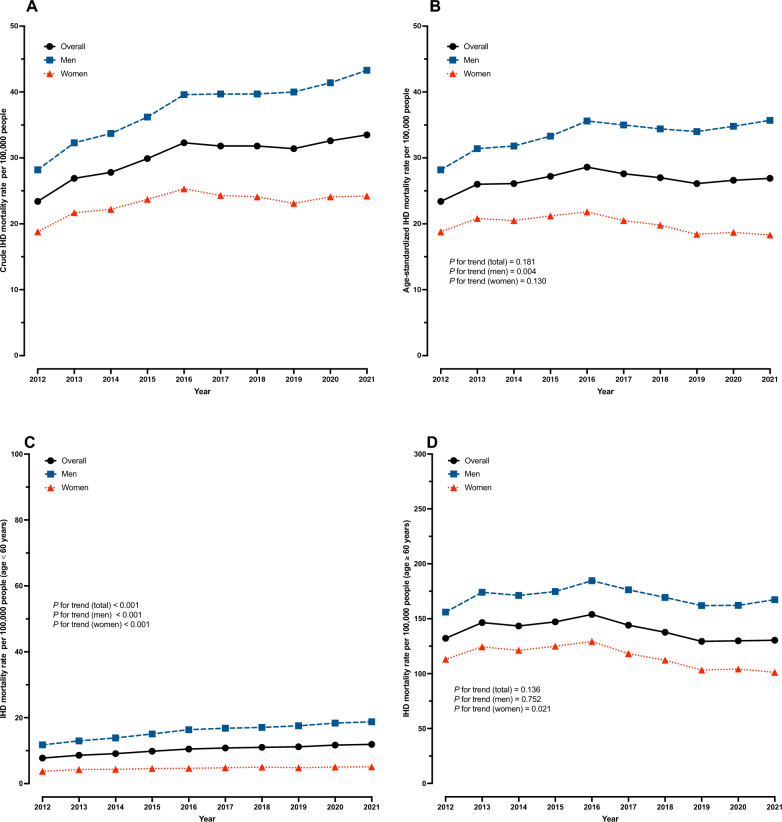
Trends in mortality rates for IHD (per 100,000 people) in Thailand from 2012 to 2021, (**A**) crude mortality rate, (**B**) age-standardized mortality rate, (**C**) mortality rate in people aged < 60 years, (**D**) mortality rate in people aged ≥ 60 years

The overall age-standardized IHD mortality rate in 2012 was 23.4 per 100,000 people, peaked at 28.6 per 100,000 in 2016, and reached 26.9 per 100,000 in 2021, *p* for trend = 0.181. In men, significant rising trends in age-standardized IHD mortality rate were observed, increasing by 26.6% over a decade, *p *for trend = 0.004 (Table 1). Whereas the age-standardized IHD mortality rate among women was consistent from 2012 to 2021, *p* for trend = 0.130 (Fig. 2). The IHD mortality rate among Thai people aged less than 60 has been continuously increasing from 2012 to 2021, with a rise of 59.6% in men and 36.1% in women, while the rate for those aged 60 and over remained steady (Supplementary Table 2).

## Discussion

Our study indicates that there has been a consistent pattern in the age-standardized hospital admission rates for IHD among Thai people from 2012 to 2021, with a decrease in rates observed among women. However, for people under the age of 60, we observed an increasing trend in hospital admission rates for IHD. As for the IHD mortality rate, we found that the age-standardized mortality rate has increased among men but not among women. Nevertheless, among men and women aged less than 60, IHD mortality rates substantially increased over the past decade.

Over a decade, we discovered that the hospital admission rate for IHD among the Thai population remained steady and showed a decreasing trend in women. This can be attributed to improvements in the healthcare system and effective management of risk factors for IHD in certain populations. Thailand has had universal health coverage since 2002, providing free access to essential healthcare services, including prevention and treatment, to its entire population [36, 37]. This allows individuals experiencing emergency conditions like IHD to seek medical attention at any hospital in Thailand [38, 39]. Collaborating with organizations like the Thai HTN Society and the MoPH, guidelines have been developed and promoted for managing HTN and diabetes, which are major risk factors for IHD. These guidelines are to be followed in all outpatient clinics nationwide to improve care and management for people with these conditions [40–43]. Nevertheless, the hospital admission rate alone cannot fully represent the prevalence of IHD in Thailand. Higher hospitalization rates may indicate higher prevalence, but advancements in treatment and public health measures could also reduce hospitalization rates despite increasing IHD prevalence.

According to our findings, the rates of hospitalization and mortality due to IHD among the Thai population have not improved significantly in the past decade. Although the overall hospital admission rates have decreased, both hospitalization and mortality rates due to IHD have continued to rise among men. This can be attributed to various risk factors associated with IHD, such as metabolic and lifestyle factors, and men are more susceptible to these risk factors than women [9–11, 13, 44–46]. According to the GBD study, in southeast Asia, the percentage of disability-adjusted life years (DALYs) due to IHD was attributable to high systolic BP, accounting for 58.2%, which was the highest among CVD risk factors. At the same time, high LDL cholesterol and smoking had attributable DALYs of 48.2% and 27.6%, respectively [47].

In Thailand, the prevalence of HTN among Thai adults increased from 21.4% in 2009 to 25.4% in 2019 (26.7% in men and 24.2% in women), with approximately 14 million Thai adults facing HTN [7–9]. A recent nationwide study also found that only 50% of Thai people with HTN can control their BP to less than 140/90 mmHg during their last two follow-up visits, and men are more likely to have uncontrolled BP than women [44]. Moreover, the study also found that only one-third of Thai people with HTN had LDL cholesterol < 100 mg/dL [44]. However, to gain a better understanding of the epidemiology of IHD among high-risk Thai populations such as those with HTN, further investigation is needed.

There is well-documented evidence that patients with coexisting HTN and T2D tended to have a higher risk for IHD [45, 48]. A nationwide study of Thai people with T2D from 2011 to 2018 found that only one-third of patients had glycemic control and also pointed out that men with T2D tend to have poorer optimal BP compared to women [46]. Cigarette smoking is the major lifestyle risk factor for IHD among Thai men. Regarding the NHES report in Thailand, the prevalence of current smoking cigarettes among men (35.5%) is substantially higher than among women (2.8%) [9].

It has been observed that hospital admission and mortality rates for IHD have increased among people under the age of 60 over the past decade, while a significant reduction in hospitalization rates and a decreasing trend in mortality rates due to IHD has been seen among people aged 60 and above. These findings could be due to the lack of awareness of IHD risk factors among different age groups. For example, according to the NHES reports, the percentage of Thai hypertensive patients under the age of 60 who were neither diagnosed nor treated was about 57% and 60% in 2009 and 2019, respectively, while the percentage was 37% and 35% for those aged 60 and above in 2009 [8] and 2019 [9]. This finding underscores the need for urgent and concerted efforts towards improving healthcare services that prioritize health promotion, prevention, and treatment, particularly among young people in Thailand. Such measures are expected to be crucial in mitigating the risk of IHD and reducing premature mortality [49–51].

### Limitation

We obtained the data on IHD cases from secondary databases, which limited our ability to perform age adjustment with precision. We could only perform age-standardization into two categories: below 60 and 60 and above. Moreover, patients with unrecognized or silent myocardial infarction who did not exhibit significant symptoms might not have gone to the hospital. Therefore, hospital admission rates could be underrepresented.

### Supplementary Information

Supplementary Material 1.

## Data Availability

The datasets generated and/or analyzed during the current study are available at https://spd.moph.go.th/public-health-statistics and https://spd.moph.go.th/illness-report/.

## Abbreviations

BP: Blood pressure
CVD: Cardiovascular disease
GBD: Global burden disease
HTN: Hypertension
ICD-10: International Statistical Classification of Diseases and Related Health Problems, Tenth revision
IHD: Ischemic heart disease
MoPH: Ministry of Public Health
NHES: National Health Examination Survey
T2D: Type 2 diabetes
