# Supplementary material for: Hospital admission and mortality rates for ischemic heart disease in Thailand: 2012–2021
Source: BMC Res Notes. 2024 May 19;17:142. doi: 10.1186/s13104-024-06803-x (PMC11102613; doi:10.1186/s13104-024-06803-x)
Supplement: Supplementary file 1 — Supplementary Material 1. [file 13104_2024_6803_MOESM1_ESM.docx]

**Supplementary data**

**Hospital admission and mortality rates for ischemic heart disease in Thailand: 2012-2021**

*Boonsub Sakboonyarat^1^ and *Ram Rangsin^1^*

^1^Department of Military and Community Medicine, Phramongkutklao College of Medicine, Bangkok 10400, Thailand

***Corresponding author:**

Ram Rangsin, MD, MPH DrPH

Professor of Epidemiology

Department of Military and Community Medicine, Phramongkutklao College of Medicine, Bangkok 10400, Thailand

**Supplementary Table 1.** Age-specific hospital admission rates for ischemic heart disease (IHD) in Thailand from 2012 to 2021

| **Year** | **Hospital admission rate for IHD per 100,000 people**  **(Age <60 years)** | | | **Hospital admission rate for IHD per 100,000 people**  **(Age ≥ 60 years)** | | |
| --- | --- | --- | --- | --- | --- | --- |
|  | **Overall** | **Men** | **Women** | **Overall** | **Men** | **Women** |
| 2012 | 128.0 | 158.0 | 98.1 | 2500.6 | 2828.4 | 2238.2 |
| 2013 | 118.1 | 147.8 | 88.6 | 2480.9 | 2863.0 | 2175.2 |
| 2014 | 111.7 | 142.3 | 81.2 | 2233.5 | 2589.8 | 1948.8 |
| 2015 | 169.6 | 239.8 | 99.8 | 2567.3 | 3026.5 | 2199.1 |
| 2016 | 152.1 | 210.8 | 93.7 | 2462.5 | 2898.4 | 2112.0 |
| 2017 | 147.2 | 201.3 | 93.3 | 2394.0 | 2876.4 | 2006.8 |
| 2018 | 152.2 | 209.1 | 95.5 | 2370.5 | 2890.2 | 1953.8 |
| 2019 | 160.3 | 222.1 | 98.7 | 2356.3 | 2911.0 | 1912.1 |
| 2020 | 164.1 | 230.2 | 98.3 | 2292.3 | 2870.0 | 1832.4 |
| 2021 | 154.4 | 216.3 | 92.8 | 2024.0 | 2577.8 | 1585.9 |
| **% change, 2012-21** | 20.6 | 36.9 | -5.4 | -19.1 | -8.9 | -29.1 |
| ***P* for trend** | 0.027 | 0.017 | 0.390 | 0.038 | 0.712 | 0.001 |

**Supplementary Table 2.** Age-specific ischemic heart disease (IHD) mortality rate in Thailand from 2012 to 2021

| **Year** | **IHD mortality rate per 100,000 people (Age <60 years)** | | | **IHD mortality rate per 100,000 people (Age ≥ 60 years)** | | |
| --- | --- | --- | --- | --- | --- | --- |
|  | **Overall** | **Men** | **Women** | **Overall** | **Men** | **Women** |
| 2012 | 7.7 | 11.8 | 3.7 | 132.2 | 156.0 | 113.1 |
| 2013 | 8.6 | 13.0 | 4.3 | 146.5 | 174.1 | 124.4 |
| 2014 | 9.1 | 13.9 | 4.3 | 143.4 | 171.2 | 121.2 |
| 2015 | 9.8 | 15.1 | 4.6 | 147.1 | 174.8 | 124.9 |
| 2016 | 10.5 | 16.4 | 4.6 | 153.9 | 184.6 | 129.3 |
| 2017 | 10.8 | 16.8 | 4.8 | 144.1 | 176.4 | 118.2 |
| 2018 | 11.0 | 17.0 | 5.0 | 137.7 | 169.4 | 112.2 |
| 2019 | 11.2 | 17.6 | 4.8 | 129.4 | 162.0 | 103.3 |
| 2020 | 11.7 | 18.4 | 5.0 | 129.9 | 162.2 | 104.2 |
| 2021 | 11.9 | 18.8 | 5.1 | 130.4 | 167.3 | 101.2 |
| **% change, 2012-21** | 53.9 | 59.6 | 36.1 | -1.3 | 7.2 | -10.5 |
| ***P* for trend** | <0.001 | <0.001 | <0.001 | 0.136 | 0.752 | 0.021 |
